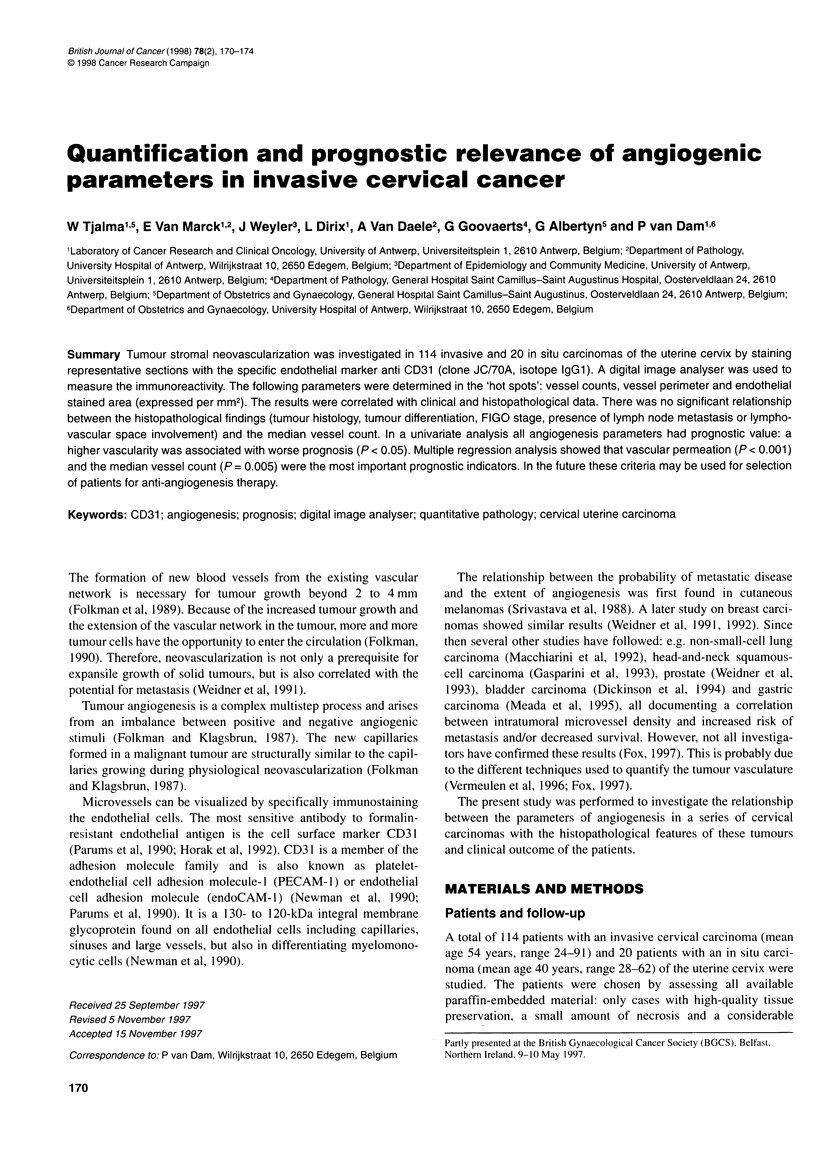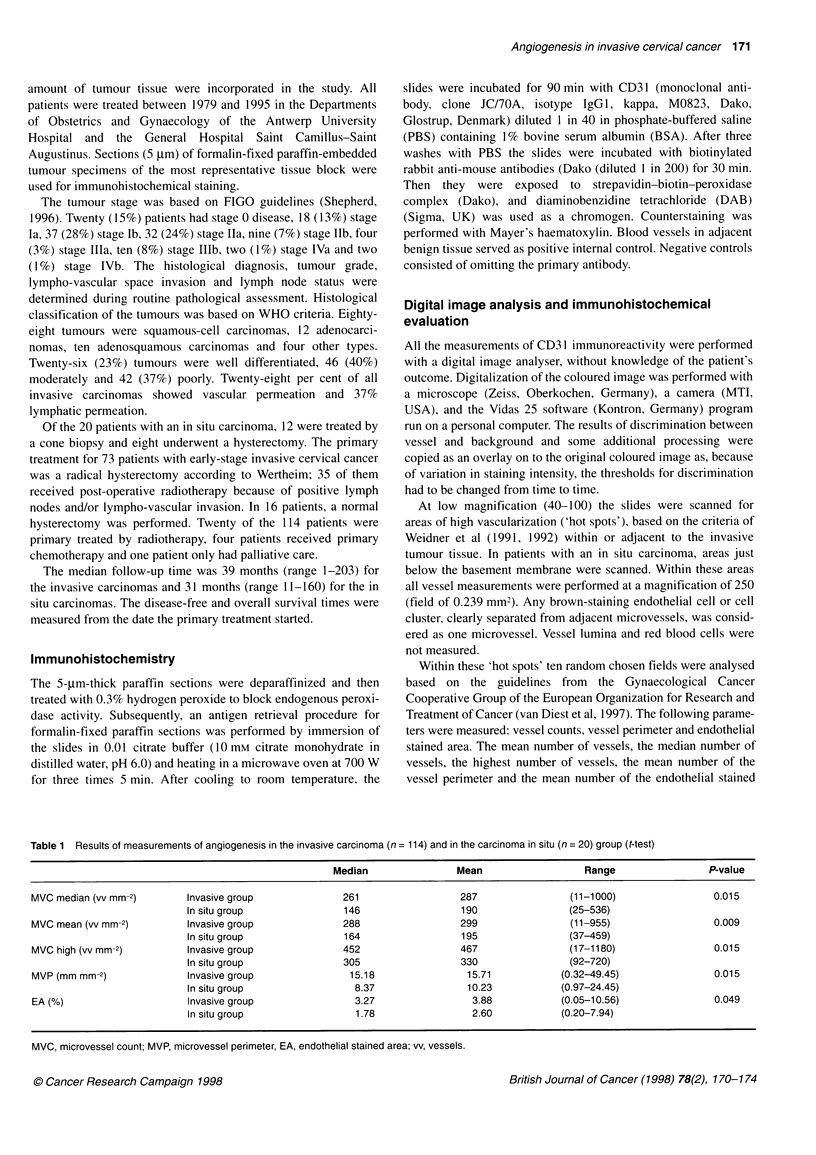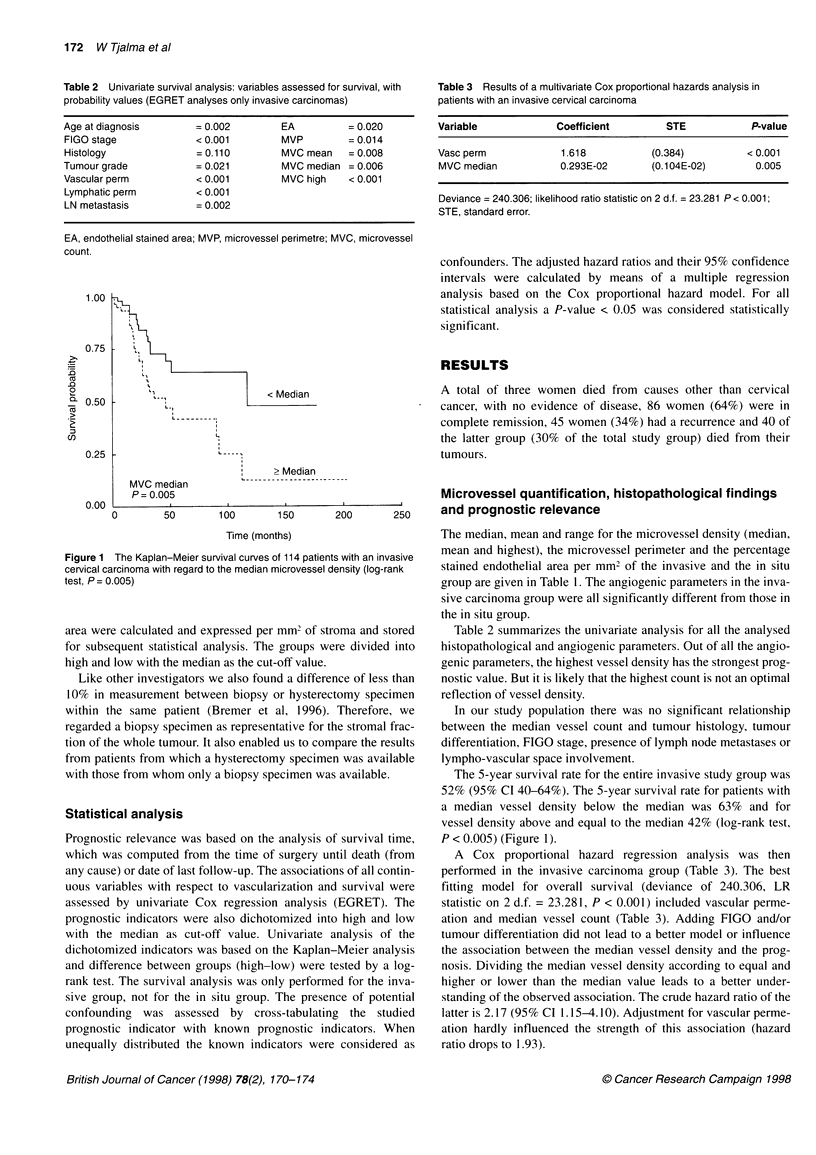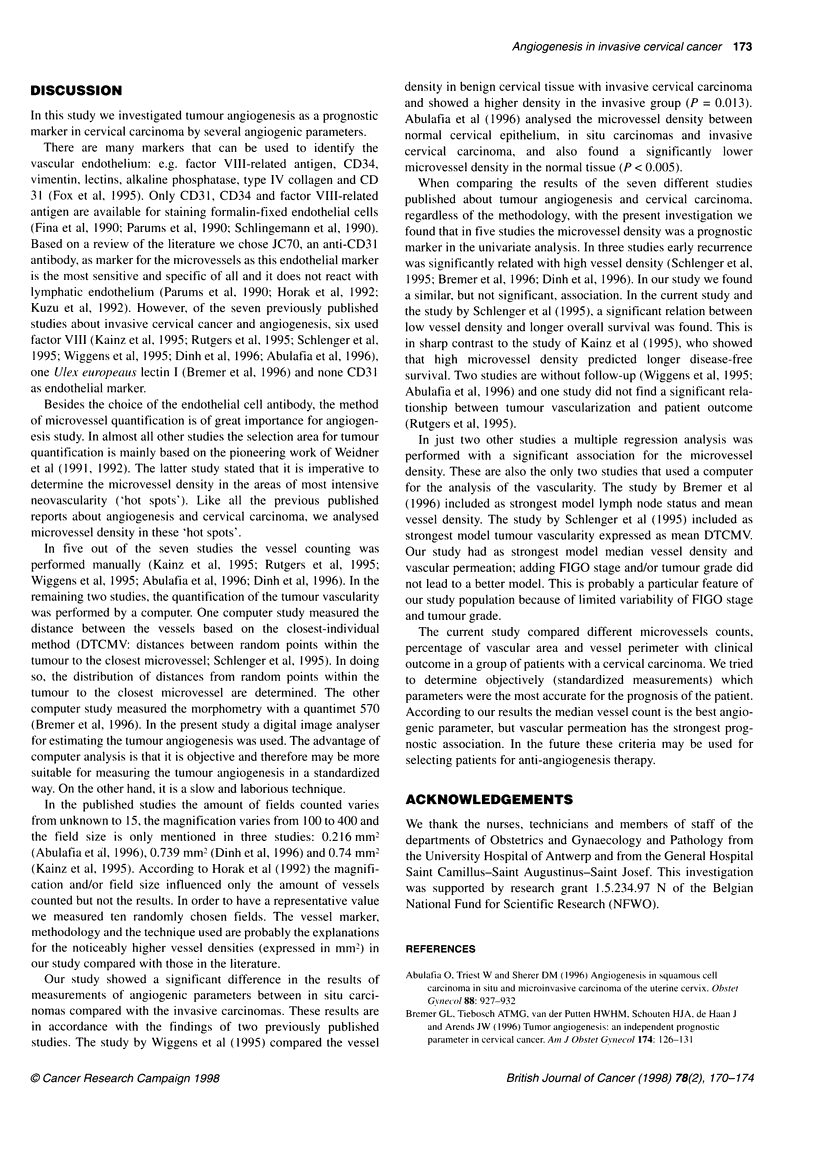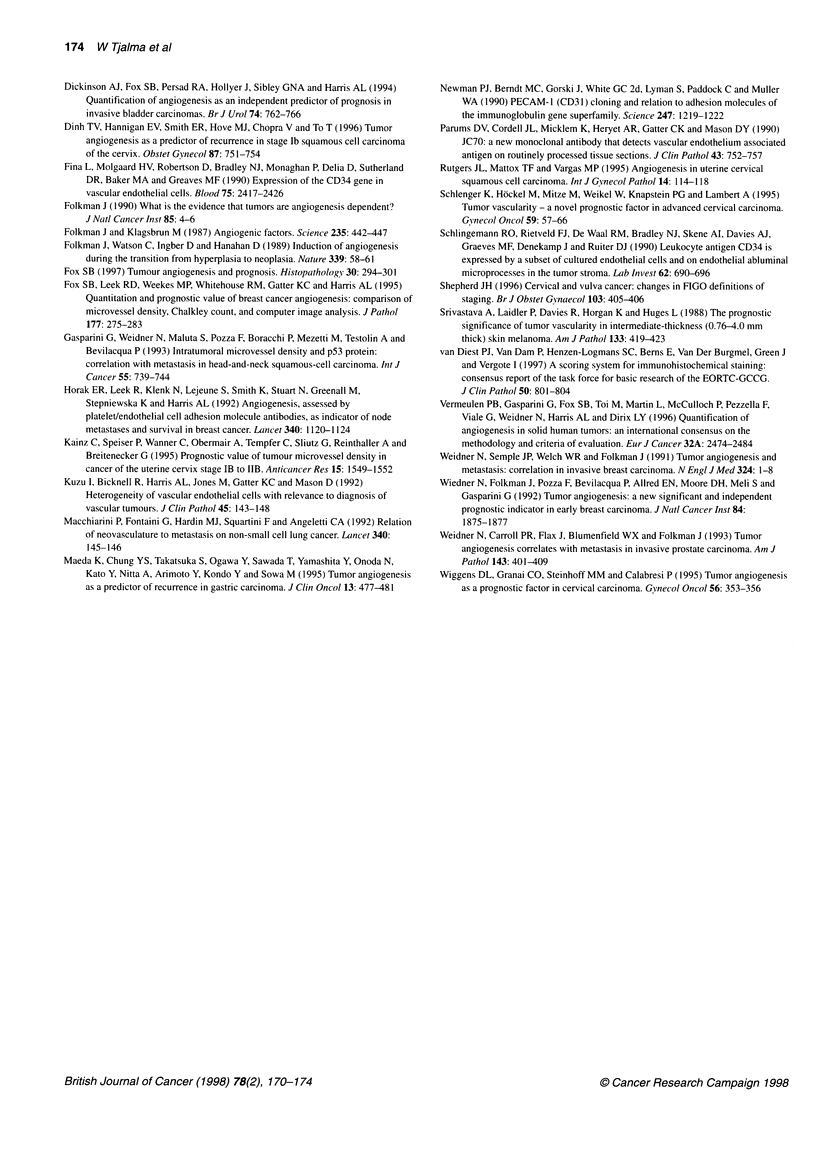# Quantification and prognostic relevance of angiogenic parameters in invasive cervical cancer.

**DOI:** 10.1038/bjc.1998.460

**Published:** 1998-07

**Authors:** W. Tjalma, E. Van Marck, J. Weyler, L. Dirix, A. Van Daele, G. Goovaerts, G. Albertyn, P. van Dam

**Affiliations:** Laboratory of Cancer Research and Clinical Oncology, University of Antwerp, and General Hospital Saint Camillus-Saint Augustinus, Belgium.

## Abstract

Tumour stromal neovascularization was investigated in 114 invasive and 20 in situ carcinomas of the uterine cervix by staining representative sections with the specific endothelial marker anti CD31 (clone JC/70A, isotope IgG1). A digital image analyser was used to measure the immunoreactivity. The following parameters were determined in the 'hot spots': vessel counts, vessel perimeter and endothelial stained area (expressed per mm2). The results were correlated with clinical and histopathological data. There was no significant relationship between the histopathological findings (tumour histology, tumour differentiation, FIGO stage, presence of lymph node metastasis or lymphovascular space involvement) and the median vessel count. In a univariate analysis all angiogenesis parameters had prognostic value: a higher vascularity was associated with worse prognosis (P < 0.05). Multiple regression analysis showed that vascular permeation (P < 0.001) and the median vessel count (P = 0.005) were the most important prognostic indicators. In the future these criteria may be used for selection of patients for anti-angiogenesis therapy.


British Journal of Cancer (1998) 78(2), 170–174
© 1998 Cancer Research Campaign

### Quantification and prognostic relevance of angiogenic parameters in invasive cervical cancer

**W Tjalma[1,5], E Van Marck[1,2], J Weyler[3], L Dirix[1], A Van Daele[2], G Goovaerts[4], G Albertyn[5] and P van Dam[1,6]**

[1]Laboratory of Cancer Research and Clinical Oncology, University of Antwerp, Universiteitsplein 1, 2610 Antwerp, Belgium; [2]Department of Pathology, University Hospital of Antwerp, Wilrijkstraat 10, 2650 Edegem, Belgium; [3]Department of Epidemiology and Community Medicine, University of Antwerp, Universiteitsplein 1, 2610 Antwerp, Belgium; [4]Department of Pathology, General Hospital Saint Camillus–Saint Augustinus Hospital, Oosterveldlaan 24, 2610 Antwerp, Belgium; [5]Department of Obstetrics and Gynaecology, General Hospital Saint Camillus–Saint Augustinus, Oosterveldlaan 24, 2610 Antwerp, Belgium; [6]Department of Obstetrics and Gynaecology, University Hospital of Antwerp, Wilrijkstraat 10, 2650 Edegem, Belgium

**Summary** Tumour stromal neovascularization was investigated in 114 invasive and 20 in situ carcinomas of the uterine cervix by staining representative sections with the specific endothelial marker anti CD31 (clone JC/70A, isotope IgG1). A digital image analyser was used to measure the immunoreactivity. The following parameters were determined in the 'hot spots': vessel counts, vessel perimeter and endothelial stained area (expressed per mm$^2$). The results were correlated with clinical and histopathological data. There was no significant relationship between the histopathological findings (tumour histology, tumour differentiation, FIGO stage, presence of lymph node metastasis or lympho-vascular space involvement) and the median vessel count. In a univariate analysis all angiogenesis parameters had prognostic value: a higher vascularity was associated with worse prognosis ($P < 0.05$). Multiple regression analysis showed that vascular permeation ($P < 0.001$) and the median vessel count ($P = 0.005$) were the most important prognostic indicators. In the future these criteria may be used for selection of patients for anti-angiogenesis therapy.

**Keywords:** CD31; angiogenesis; prognosis; digital image analyser; quantitative pathology; cervical uterine carcinoma

The formation of new blood vessels from the existing vascular network is necessary for tumour growth beyond 2 to 4 mm (Folkman et al, 1989). Because of the increased tumour growth and the extension of the vascular network in the tumour, more and more tumour cells have the opportunity to enter the circulation (Folkman, 1990). Therefore, neovascularization is not only a prerequisite for expansile growth of solid tumours, but is also correlated with the potential for metastasis (Weidner et al, 1991).

Tumour angiogenesis is a complex multistep process and arises from an imbalance between positive and negative angiogenic stimuli (Folkman and Klagsbrun, 1987). The new capillaries formed in a malignant tumour are structurally similar to the capillaries growing during physiological neovascularization (Folkman and Klagsbrun, 1987).

Microvessels can be visualized by specifically immunostaining the endothelial cells. The most sensitive antibody to formalin-resistant endothelial antigen is the cell surface marker CD31 (Parums et al, 1990; Horak et al, 1992). CD31 is a member of the adhesion molecule family and is also known as platelet-endothelial cell adhesion molecule-1 (PECAM-1) or endothelial cell adhesion molecule (endoCAM-1) (Newman et al, 1990; Parums et al, 1990). It is a 130- to 120-kDa integral membrane glycoprotein found on all endothelial cells including capillaries, sinuses and large vessels, but also in differentiating myelomonocytic cells (Newman et al, 1990).

*Received 25 September 1997*
*Revised 5 November 1997*
*Accepted 15 November 1997*

*Correspondence to:* P van Dam, Wilrijkstraat 10, 2650 Edegem, Belgium

The relationship between the probability of metastatic disease and the extent of angiogenesis was first found in cutaneous melanomas (Srivastava et al, 1988). A later study on breast carcinomas showed similar results (Weidner et al, 1991, 1992). Since then several other studies have followed: e.g. non-small-cell lung carcinoma (Macchiarini et al, 1992), head-and-neck squamous-cell carcinoma (Gasparini et al, 1993), prostate (Weidner et al, 1993), bladder carcinoma (Dickinson et al, 1994) and gastric carcinoma (Meada et al, 1995), all documenting a correlation between intratumoral microvessel density and increased risk of metastasis and/or decreased survival. However, not all investigators have confirmed these results (Fox, 1997). This is probably due to the different techniques used to quantify the tumour vasculature (Vermeulen et al, 1996; Fox, 1997).

The present study was performed to investigate the relationship between the parameters of angiogenesis in a series of cervical carcinomas with the histopathological features of these tumours and clinical outcome of the patients.

### MATERIALS AND METHODS

### Patients and follow-up

A total of 114 patients with an invasive cervical carcinoma (mean age 54 years, range 24–91) and 20 patients with an in situ carcinoma (mean age 40 years, range 28–62) of the uterine cervix were studied. The patients were chosen by assessing all available paraffin-embedded material: only cases with high-quality tissue preservation, a small amount of necrosis and a considerable

Partly presented at the British Gynaecological Cancer Society (BGCS), Belfast, Northern Ireland, 9–10 May 1997.

amount of tumour tissue were incorporated in the study. All patients were treated between 1979 and 1995 in the Departments of Obstetrics and Gynaecology of the Antwerp University Hospital and the General Hospital Saint Camillus–Saint Augustinus. Sections (5 μm) of formalin-fixed paraffin-embedded tumour specimens of the most representative tissue block were used for immunohistochemical staining.

The tumour stage was based on FIGO guidelines (Shepherd, 1996). Twenty (15%) patients had stage 0 disease, 18 (13%) stage Ia, 37 (28%) stage Ib, 32 (24%) stage IIa, nine (7%) stage IIb, four (3%) stage IIIa, ten (8%) stage IIIb, two (1%) stage IVa and two (1%) stage IVb. The histological diagnosis, tumour grade, lympho-vascular space invasion and lymph node status were determined during routine pathological assessment. Histological classification of the tumours was based on WHO criteria. Eighty-eight tumours were squamous-cell carcinomas, 12 adenocarcinomas, ten adenosquamous carcinomas and four other types. Twenty-six (23%) tumours were well differentiated, 46 (40%) moderately and 42 (37%) poorly. Twenty-eight per cent of all invasive carcinomas showed vascular permeation and 37% lymphatic permeation.

Of the 20 patients with an in situ carcinoma, 12 were treated by a cone biopsy and eight underwent a hysterectomy. The primary treatment for 73 patients with early-stage invasive cervical cancer was a radical hysterectomy according to Wertheim; 35 of them received post-operative radiotherapy because of positive lymph nodes and/or lympho-vascular invasion. In 16 patients, a normal hysterectomy was performed. Twenty of the 114 patients were primary treated by radiotherapy, four patients received primary chemotherapy and one patient only had palliative care.

The median follow-up time was 39 months (range 1–203) for the invasive carcinomas and 31 months (range 11–160) for the in situ carcinomas. The disease-free and overall survival times were measured from the date the primary treatment started.

### Immunohistochemistry

The 5-μm-thick paraffin sections were deparaffinized and then treated with 0.3% hydrogen peroxide to block endogenous peroxidase activity. Subsequently, an antigen retrieval procedure for formalin-fixed paraffin sections was performed by immersion of the slides in 0.01 citrate buffer (10 mM citrate monohydrate in distilled water, pH 6.0) and heating in a microwave oven at 700 W for three times 5 min. After cooling to room temperature, the slides were incubated for 90 min with CD31 (monoclonal antibody, clone JC/70A, isotype IgG1, kappa, M0823, Dako, Glostrup, Denmark) diluted 1 in 40 in phosphate-buffered saline (PBS) containing 1% bovine serum albumin (BSA). After three washes with PBS the slides were incubated with biotinylated rabbit anti-mouse antibodies (Dako (diluted 1 in 200) for 30 min. Then they were exposed to strepavidin–biotin–peroxidase complex (Dako), and diaminobenzidine tetrachloride (DAB) (Sigma, UK) was used as a chromogen. Counterstaining was performed with Mayer's haematoxylin. Blood vessels in adjacent benign tissue served as positive internal control. Negative controls consisted of omitting the primary antibody.

### Digital image analysis and immunohistochemical evaluation

All the measurements of CD31 immunoreactivity were performed with a digital image analyser, without knowledge of the patient's outcome. Digitalization of the coloured image was performed with a microscope (Zeiss, Oberkochen, Germany), a camera (MTI, USA), and the Vidas 25 software (Kontron, Germany) program run on a personal computer. The results of discrimination between vessel and background and some additional processing were copied as an overlay on to the original coloured image as, because of variation in staining intensity, the thresholds for discrimination had to be changed from time to time.

At low magnification (40–100) the slides were scanned for areas of high vascularization ('hot spots'), based on the criteria of Weidner et al (1991, 1992) within or adjacent to the invasive tumour tissue. In patients with an in situ carcinoma, areas just below the basement membrane were scanned. Within these areas all vessel measurements were performed at a magnification of 250 (field of 0.239 mm$^2$). Any brown-staining endothelial cell or cell cluster, clearly separated from adjacent microvessels, was considered as one microvessel. Vessel lumina and red blood cells were not measured.

Within these 'hot spots' ten random chosen fields were analysed based on the guidelines from the Gynaecological Cancer Cooperative Group of the European Organization for Research and Treatment of Cancer (van Diest et al, 1997). The following parameters were measured: vessel counts, vessel perimeter and endothelial stained area. The mean number of vessels, the median number of vessels, the highest number of vessels, the mean number of the vessel perimeter and the mean number of the endothelial stained

**Table 1** Results of measurements of angiogenesis in the invasive carcinoma ($n$ = 114) and in the carcinoma in situ ($n$ = 20) group ($t$-test)

| | | Median | Mean | Range | $P$-value |
|---|---|---|---|---|---|
| MVC median (vv mm$^{-2}$) | Invasive group | 261 | 287 | (11–1000) | 0.015 |
| | In situ group | 146 | 190 | (25–536) | |
| MVC mean (vv mm$^{-2}$) | Invasive group | 288 | 299 | (11–955) | 0.009 |
| | In situ group | 164 | 195 | (37–459) | |
| MVC high (vv mm$^{-2}$) | Invasive group | 452 | 467 | (17–1180) | 0.015 |
| | In situ group | 305 | 330 | (92–720) | |
| MVP (mm mm$^{-2}$) | Invasive group | 15.18 | 15.71 | (0.32–49.45) | 0.015 |
| | In situ group | 8.37 | 10.23 | (0.97–24.45) | |
| EA (%) | Invasive group | 3.27 | 3.88 | (0.05–10.56) | 0.049 |
| | In situ group | 1.78 | 2.60 | (0.20–7.94) | |

MVC, microvessel count; MVP, microvessel perimeter, EA, endothelial stained area; vv, vessels.

© Cancer Research Campaign 1998

Table 2 Univariate survival analysis: variables assessed for survival, with probability values (EGRET analyses only invasive carcinomas)

| | | | |
|---|---|---|---|
| Age at diagnosis | = 0.002 | EA | = 0.020 |
| FIGO stage | < 0.001 | MVP | = 0.014 |
| Histology | = 0.110 | MVC mean | = 0.008 |
| Tumour grade | = 0.021 | MVC median | = 0.006 |
| Vascular perm | < 0.001 | MVC high | < 0.001 |
| Lymphatic perm | < 0.001 | | |
| LN metastasis | = 0.002 | | |

EA, endothelial stained area; MVP, microvessel perimetre; MVC, microvessel count.

Figure 1 The Kaplan–Meier survival curves of 114 patients with an invasive cervical carcinoma with regard to the median microvessel density (log-rank test, $P = 0.005$)

area were calculated and expressed per mm$^2$ of stroma and stored for subsequent statistical analysis. The groups were divided into high and low with the median as the cut-off value.

Like other investigators we also found a difference of less than 10% in measurement between biopsy or hysterectomy specimen within the same patient (Bremer et al, 1996). Therefore, we regarded a biopsy specimen as representative for the stromal fraction of the whole tumour. It also enabled us to compare the results from patients from which a hysterectomy specimen was available with those from whom only a biopsy specimen was available.

### Statistical analysis

Prognostic relevance was based on the analysis of survival time, which was computed from the time of surgery until death (from any cause) or date of last follow-up. The associations of all continuous variables with respect to vascularization and survival were assessed by univariate Cox regression analysis (EGRET). The prognostic indicators were also dichotomized into high and low with the median as cut-off value. Univariate analysis of the dichotomized indicators was based on the Kaplan–Meier analysis and difference between groups (high–low) were tested by a log-rank test. The survival analysis was only performed for the invasive group, not for the in situ group. The presence of potential confounding was assessed by cross-tabulating the studied prognostic indicator with known prognostic indicators. When unequally distributed the known indicators were considered as confounders. The adjusted hazard ratios and their 95% confidence intervals were calculated by means of a multiple regression analysis based on the Cox proportional hazard model. For all statistical analysis a $P$-value < 0.05 was considered statistically significant.

Table 3 Results of a multivariate Cox proportional hazards analysis in patients with an invasive cervical carcinoma

| Variable | Coefficient | STE | *P*-value |
|---|---|---|---|
| Vasc perm | 1.618 | (0.384) | < 0.001 |
| MVC median | 0.293E-02 | (0.104E-02) | 0.005 |

Deviance = 240.306; likelihood ratio statistic on 2 d.f. = 23.281 $P < 0.001$; STE, standard error.

### RESULTS

A total of three women died from causes other than cervical cancer, with no evidence of disease, 86 women (64%) were in complete remission, 45 women (34%) had a recurrence and 40 of the latter group (30% of the total study group) died from their tumours.

### Microvessel quantification, histopathological findings and prognostic relevance

The median, mean and range for the microvessel density (median, mean and highest), the microvessel perimeter and the percentage stained endothelial area per mm$^2$ of the invasive and the in situ group are given in Table 1. The angiogenic parameters in the invasive carcinoma group were all significantly different from those in the in situ group.

Table 2 summarizes the univariate analysis for all the analysed histopathological and angiogenic parameters. Out of all the angiogenic parameters, the highest vessel density has the strongest prognostic value. But it is likely that the highest count is not an optimal reflection of vessel density.

In our study population there was no significant relationship between the median vessel count and tumour histology, tumour differentiation, FIGO stage, presence of lymph node metastases or lympho-vascular space involvement.

The 5-year survival rate for the entire invasive study group was 52% (95% CI 40–64%). The 5-year survival rate for patients with a median vessel density below the median was 63% and for vessel density above and equal to the median 42% (log-rank test, $P < 0.005$) (Figure 1).

A Cox proportional hazard regression analysis was then performed in the invasive carcinoma group (Table 3). The best fitting model for overall survival (deviance of 240.306, LR statistic on 2 d.f. = 23.281, $P < 0.001$) included vascular permeation and median vessel count (Table 3). Adding FIGO and/or tumour differentiation did not lead to a better model or influence the association between the median vessel density and the prognosis. Dividing the median vessel density according to equal and higher or lower than the median value leads to a better understanding of the observed association. The crude hazard ratio of the latter is 2.17 (95% CI 1.15–4.10). Adjustment for vascular permeation hardly influenced the strength of this association (hazard ratio drops to 1.93).

 © Cancer Research Campaign 1998

### DISCUSSION

In this study we investigated tumour angiogenesis as a prognostic marker in cervical carcinoma by several angiogenic parameters.

There are many markers that can be used to identify the vascular endothelium: e.g. factor VIII-related antigen, CD34, vimentin, lectins, alkaline phosphatase, type IV collagen and CD 31 (Fox et al, 1995). Only CD31, CD34 and factor VIII-related antigen are available for staining formalin-fixed endothelial cells (Fina et al, 1990; Parums et al, 1990; Schlingemann et al, 1990). Based on a review of the literature we chose JC70, an anti-CD31 antibody, as marker for the microvessels as this endothelial marker is the most sensitive and specific of all and it does not react with lymphatic endothelium (Parums et al, 1990; Horak et al, 1992; Kuzu et al, 1992). However, of the seven previously published studies about invasive cervical cancer and angiogenesis, six used factor VIII (Kainz et al, 1995; Rutgers et al, 1995; Schlenger et al, 1995; Wiggens et al, 1995; Dinh et al, 1996; Abulafia et al, 1996), one *Ulex europeaus* lectin I (Bremer et al, 1996) and none CD31 as endothelial marker.

Besides the choice of the endothelial cell antibody, the method of microvessel quantification is of great importance for angiogenesis study. In almost all other studies the selection area for tumour quantification is mainly based on the pioneering work of Weidner et al (1991, 1992). The latter study stated that it is imperative to determine the microvessel density in the areas of most intensive neovascularity ('hot spots'). Like all the previous published reports about angiogenesis and cervical carcinoma, we analysed microvessel density in these 'hot spots'.

In five out of the seven studies the vessel counting was performed manually (Kainz et al, 1995; Rutgers et al, 1995; Wiggens et al, 1995; Abulafia et al, 1996; Dinh et al, 1996). In the remaining two studies, the quantification of the tumour vascularity was performed by a computer. One computer study measured the distance between the vessels based on the closest-individual method (DTCMV: distances between random points within the tumour to the closest microvessel; Schlenger et al, 1995). In doing so, the distribution of distances from random points within the tumour to the closest microvessel are determined. The other computer study measured the morphometry with a quantimet 570 (Bremer et al, 1996). In the present study a digital image analyser for estimating the tumour angiogenesis was used. The advantage of computer analysis is that it is objective and therefore may be more suitable for measuring the tumour angiogenesis in a standardized way. On the other hand, it is a slow and laborious technique.

In the published studies the amount of fields counted varies from unknown to 15, the magnification varies from 100 to 400 and the field size is only mentioned in three studies: 0.216 $mm^2$ (Abulafia et al, 1996), 0.739 $mm^2$ (Dinh et al, 1996) and 0.74 $mm^2$ (Kainz et al, 1995). According to Horak et al (1992) the magnification and/or field size influenced only the amount of vessels counted but not the results. In order to have a representative value we measured ten randomly chosen fields. The vessel marker, methodology and the technique used are probably the explanations for the noticeably higher vessel densities (expressed in $mm^2$) in our study compared with those in the literature.

Our study showed a significant difference in the results of measurements of angiogenic parameters between in situ carcinomas compared with the invasive carcinomas. These results are in accordance with the findings of two previously published studies. The study by Wiggens et al (1995) compared the vessel density in benign cervical tissue with invasive cervical carcinoma and showed a higher density in the invasive group ($P$ = 0.013). Abulafia et al (1996) analysed the microvessel density between normal cervical epithelium, in situ carcinomas and invasive cervical carcinoma, and also found a significantly lower microvessel density in the normal tissue ($P < 0.005$).

When comparing the results of the seven different studies published about tumour angiogenesis and cervical carcinoma, regardless of the methodology, with the present investigation we found that in five studies the microvessel density was a prognostic marker in the univariate analysis. In three studies early recurrence was significantly related with high vessel density (Schlenger et al, 1995; Bremer et al, 1996; Dinh et al, 1996). In our study we found a similar, but not significant, association. In the current study and the study by Schlenger et al (1995), a significant relation between low vessel density and longer overall survival was found. This is in sharp contrast to the study of Kainz et al (1995), who showed that high microvessel density predicted longer disease-free survival. Two studies are without follow-up (Wiggens et al, 1995; Abulafia et al, 1996) and one study did not find a significant relationship between tumour vascularization and patient outcome (Rutgers et al, 1995).

In just two other studies a multiple regression analysis was performed with a significant association for the microvessel density. These are also the only two studies that used a computer for the analysis of the vascularity. The study by Bremer et al (1996) included as strongest model lymph node status and mean vessel density. The study by Schlenger et al (1995) included as strongest model tumour vascularity expressed as mean DTCMV. Our study had as strongest model median vessel density and vascular permeation; adding FIGO stage and/or tumour grade did not lead to a better model. This is probably a particular feature of our study population because of limited variability of FIGO stage and tumour grade.

The current study compared different microvessels counts, percentage of vascular area and vessel perimeter with clinical outcome in a group of patients with a cervical carcinoma. We tried to determine objectively (standardized measurements) which parameters were the most accurate for the prognosis of the patient. According to our results the median vessel count is the best angiogenic parameter, but vascular permeation has the strongest prognostic association. In the future these criteria may be used for selecting patients for anti-angiogenesis therapy.

### ACKNOWLEDGEMENTS

We thank the nurses, technicians and members of staff of the departments of Obstetrics and Gynaecology and Pathology from the University Hospital of Antwerp and from the General Hospital Saint Camillus–Saint Augustinus–Saint Josef. This investigation was supported by research grant 1.5.234.97 N of the Belgian National Fund for Scientific Research (NFWO).

### REFERENCES

Abulafia O, Triest W and Sherer DM (1996) Angiogenesis in squamous cell carcinoma in situ and microinvasive carcinoma of the uterine cervix. *Obstet Gynecol* **88**: 927–932

Bremer GL, Tiebosch ATMG, van der Putten HWHM, Schouten HJA, de Haan J and Arends JW (1996) Tumor angiogenesis: an independent prognostic parameter in cervical cancer. *Am J Obstet Gynecol* **174**: 126–131

© Cancer Research Campaign 1998 

Dickinson AJ, Fox SB, Persad RA, Hollyer J, Sibley GNA and Harris AL (1994) Quantification of angiogenesis as an independent predictor of prognosis in invasive bladder carcinomas. *Br J Urol* **74**: 762–766

Dinh TV, Hannigan EV, Smith ER, Hove MJ, Chopra V and To T (1996) Tumor angiogenesis as a predictor of recurrence in stage Ib squamous cell carcinoma of the cervix. *Obstet Gynecol* **87**: 751–754

Fina L, Molgaard HV, Robertson D, Bradley NJ, Monaghan P, Delia D, Sutherland DR, Baker MA and Greaves MF (1990) Expression of the CD34 gene in vascular endothelial cells. *Blood* **75**: 2417–2426

Folkman J (1990) What is the evidence that tumors are angiogenesis dependent? *J Natl Cancer Inst* **85**: 4–6

Folkman J and Klagsbrun M (1987) Angiogenic factors. *Science* **235**: 442–447

Folkman J, Watson C, Ingber D and Hanahan D (1989) Induction of angiogenesis during the transition from hyperplasia to neoplasia. *Nature* **339**: 58–61

Fox SB (1997) Tumour angiogenesis and prognosis. *Histopathology* **30**: 294–301

Fox SB, Leek RD, Weekes MP, Whitehouse RM, Gatter KC and Harris AL (1995) Quantitation and prognostic value of breast cancer angiogenesis: comparison of microvessel density, Chalkley count, and computer image analysis. *J Pathol* **177**: 275–283

Gasparini G, Weidner N, Maluta S, Pozza F, Boracchi P, Mezetti M, Testolin A and Bevilacqua P (1993) Intratumoral microvessel density and p53 protein: correlation with metastasis in head-and-neck squamous-cell carcinoma. *Int J Cancer* **55**: 739–744

Horak ER, Leek R, Klenk N, Lejeune S, Smith K, Stuart N, Greenall M, Stepniewska K and Harris AL (1992) Angiogenesis, assessed by platelet/endothelial cell adhesion molecule antibodies, as indicator of node metastases and survival in breast cancer. *Lancet* **340**: 1120–1124

Kainz C, Speiser P, Wanner C, Obermair A, Tempfer C, Sliutz G, Reinthaller A and Breitenecker G (1995) Prognostic value of tumour microvessel density in cancer of the uterine cervix stage IB to IIB. *Anticancer Res* **15**: 1549–1552

Kuzu I, Bicknell R, Harris AL, Jones M, Gatter KC and Mason D (1992) Heterogeneity of vascular endothelial cells with relevance to diagnosis of vascular tumours. *J Clin Pathol* **45**: 143–148

Macchiarini P, Fontaini G, Hardin MJ, Squartini F and Angeletti CA (1992) Relation of neovasculature to metastasis on non-small cell lung cancer. *Lancet* **340**: 145–146

Maeda K, Chung YS, Takatsuka S, Ogawa Y, Sawada T, Yamashita Y, Onoda N, Kato Y, Nitta A, Arimoto Y, Kondo Y and Sowa M (1995) Tumor angiogenesis as a predictor of recurrence in gastric carcinoma. *J Clin Oncol* **13**: 477–481

Newman PJ, Berndt MC, Gorski J, White GC 2d, Lyman S, Paddock C and Muller WA (1990) PECAM-1 (CD31) cloning and relation to adhesion molecules of the immunoglobulin gene superfamily. *Science* **247**: 1219–1222

Parums DV, Cordell JL, Micklem K, Heryet AR, Gatter CK and Mason DY (1990) JC70: a new monoclonal antibody that detects vascular endothelium associated antigen on routinely processed tissue sections. *J Clin Pathol* **43**: 752–757

Rutgers JL, Mattox TF and Vargas MP (1995) Angiogenesis in uterine cervical squamous cell carcinoma. *Int J Gynecol Pathol* **14**: 114–118

Schlenger K, Höckel M, Mitze M, Weikel W, Knapstein PG and Lambert A (1995) Tumor vascularity – a novel prognostic factor in advanced cervical carcinoma. *Gynecol Oncol* **59**: 57–66

Schlingemann RO, Rietveld FJ, De Waal RM, Bradley NJ, Skene AI, Davies AJ, Graeves MF, Denekamp J and Ruiter DJ (1990) Leukocyte antigen CD34 is expressed by a subset of cultured endothelial cells and on endothelial abluminal microprocesses in the tumor stroma. *Lab Invest* **62**: 690–696

Shepherd JH (1996) Cervical and vulva cancer: changes in FIGO definitions of staging. *Br J Obstet Gynaecol* **103**: 405–406

Srivastava A, Laidler P, Davies R, Horgan K and Huges L (1988) The prognostic significance of tumor vascularity in intermediate-thickness (0.76–4.0 mm thick) skin melanoma. *Am J Pathol* **133**: 419–423

van Diest PJ, Van Dam P, Henzen-Logmans SC, Berns E, Van Der Burgmel, Green J and Vergote I (1997) A scoring system for immunohistochemical staining: consensus report of the task force for basic research of the EORTC-GCCG. *J Clin Pathol* **50**: 801–804

Vermeulen PB, Gasparini G, Fox SB, Toi M, Martin L, McCulloch P, Pezzella F, Viale G, Weidner N, Harris AL and Dirix LY (1996) Quantification of angiogenesis in solid human tumors: an international consensus on the methodology and criteria of evaluation. *Eur J Cancer* **32A**: 2474–2484

Weidner N, Semple JP, Welch WR and Folkman J (1991) Tumor angiogenesis and metastasis: correlation in invasive breast carcinoma. *N Engl J Med* **324**: 1–8

Wiedner N, Folkman J, Pozza F, Bevilacqua P, Allred EN, Moore DH, Meli S and Gasparini G (1992) Tumor angiogenesis: a new significant and independent prognostic indicator in early breast carcinoma. *J Natl Cancer Inst* **84**: 1875–1877

Weidner N, Carroll PR, Flax J, Blumenfield WX and Folkman J (1993) Tumor angiogenesis correlates with metastasis in invasive prostate carcinoma. *Am J Pathol* **143**: 401–409

Wiggens DL, Granai CO, Steinhoff MM and Calabresi P (1995) Tumor angiogenesis as a prognostic factor in cervical carcinoma. *Gynecol Oncol* **56**: 353–356

© Cancer Research Campaign 1998